# Supplementary material for: The critical role of AMPK in driving Akt activation under stress, tumorigenesis and drug resistance
Source: Nat Commun. 2018 Nov 9;9:4728. doi: 10.1038/s41467-018-07188-9 (PMC6226490; doi:10.1038/s41467-018-07188-9)
Supplement: Supplementary file 1 — Supplementary information [file 41467_2018_7188_MOESM1_ESM.pdf]

The critical role of AMPK in driving Akt activation, tumorigenesis and drug resistance

Han et al.

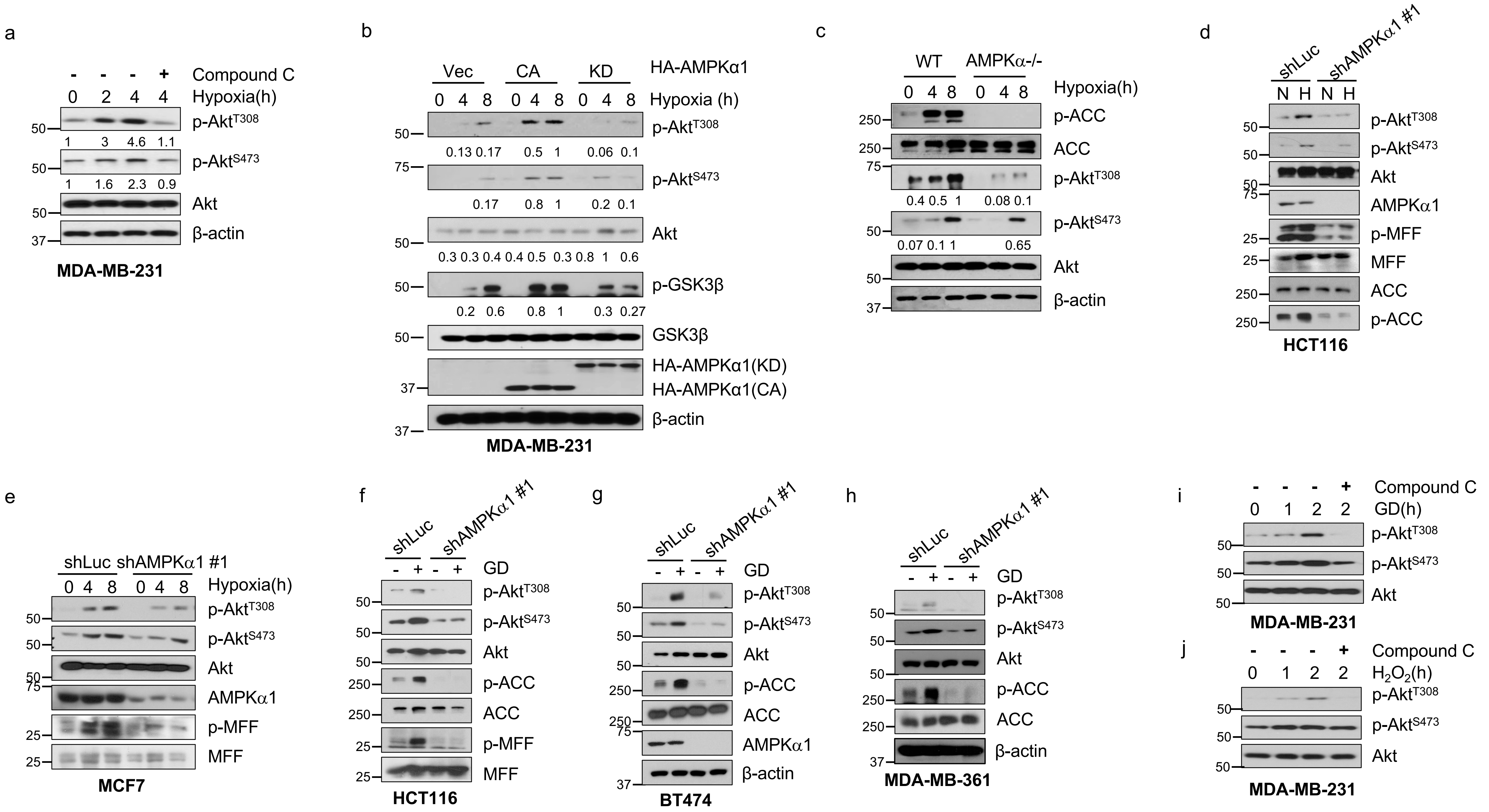

Supplementary Figure 1

Supplementary Fig. 1. AMPK and Skp2 are critical for Akt ubiquitination and activation under stresses and growth factors.

(a) Immunoblotting of MDA-MB-231 cells treated with hypoxia (1% O_2) and compound C (10 μM) for indicated time. **(b)** Immunoblotting of MDA-MB-231 cells transfected with mock (PCDNA3) and HA-AMPK (CA, KD) and challenged with 1% O2, each plasmid was transfected with 5 μg in 100mm dish. **(c)** Immunoblotting of WT and AMPKα1/α2 double knockout MEFs under hypoxia (1% O_2) for indicated time. **(d, e)** Immunoblotting of control (shLuc) and AMPKα1 knockdown (#1) HCT116, MCF7 cells under hypoxia (1% O_2) for 4 hours. **(f-h)** Immunoblotting of control (shLuc) and AMPKα1 knockdown (#1) HCT116, BT474, MDA-MB-361 cells under glucose deprivation for 2 hours. **(i)** Immunoblotting of MDA-MB-231 cells starved and treated with compound C (10 μM) and glucose deprivation (GD) for 1 and 2 hours. **(j)** Immunoblotting of MDA-MB-231 cells starved and treated with compound C (10 μM) and H_2O_2 for 1 and 2 hours.

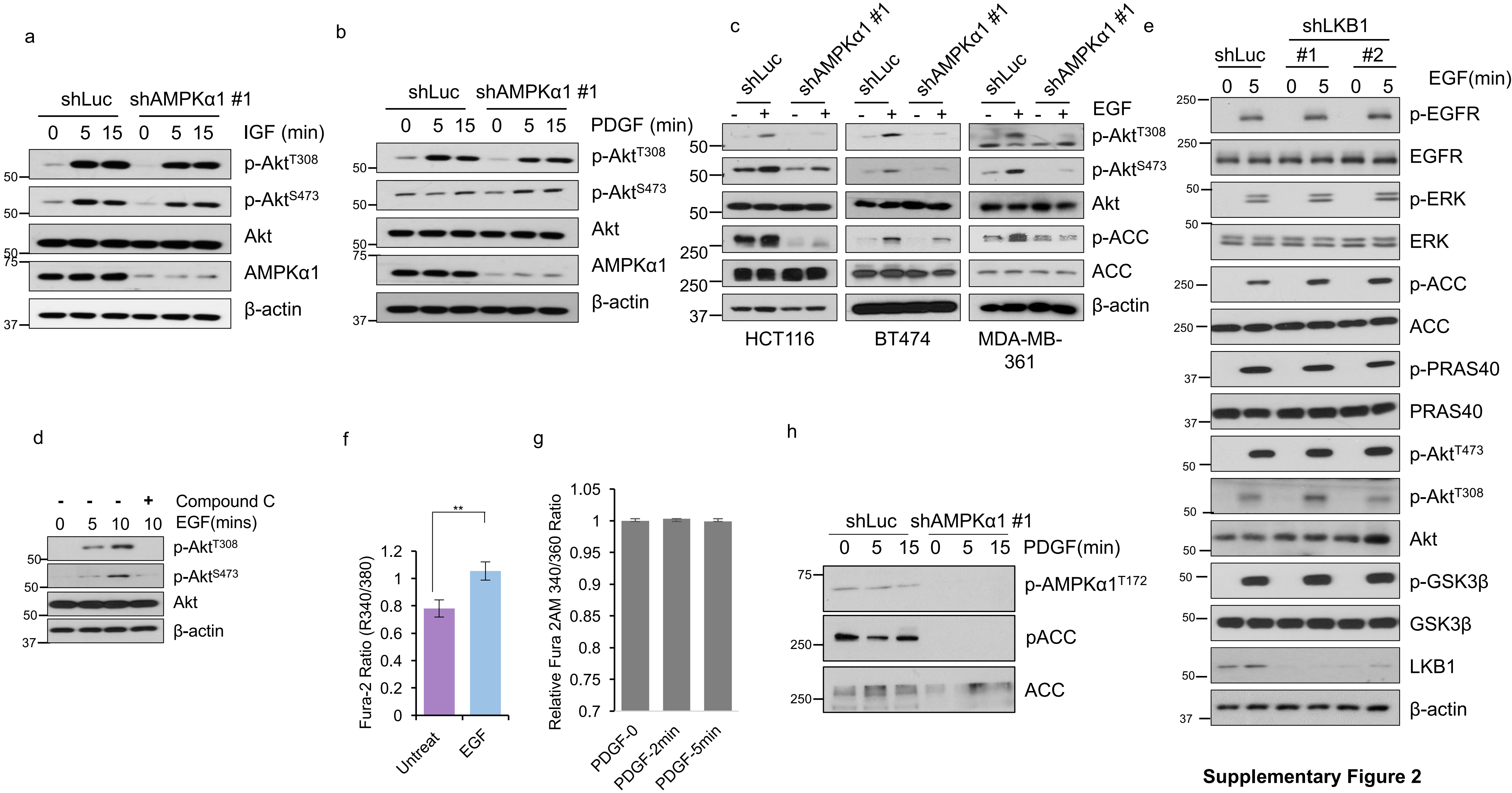

Supplementary Figure 2

Supplementary Fig. 2. IGF and PDGF fail to activate AMPK and Akt in MDA-MB-231 cells.

(a-b) Immunoblotting of control and AMPKα1 knockdown (#1) MDA-MB-231 cells serum starved and treated with IGF and PDGF for indicated time. **(c)** Immunoblotting of control (shLuc) and AMPKα1 knockdown (#1) HCT116, BT474, MDA-MB-361 cells starved for 16 hours and treated with 50 ng/ml EGF for 5 minutes. **(d)** Immunoblotting of MDA-MB-231 cells Starved and treated with compound C (10 μM) for 4 hours and EGF (50 ng/ml) for 5 minutes. **(e)** Immunoblotting of control and LKB1 knockdown (#1, #2) MDA-MB-231 cells serum starved and treated with EGF (50 ng/ml) for 5 minutes. **(f)** Relative Fura-2 AM level of MDA-MB-231 cells treated with or without EGF (50 ng/ml). **(g)** Relative Fura-2 AM level of MDA-MB-231 cells treated with or without PDGF (10 μM) for indicated time. **(h)** Immunoblotting of control and AMPKα1 knockdown (#1) MDA-MB-231 cells serum starved and treated with PDGF (10 μM) for indicated time.

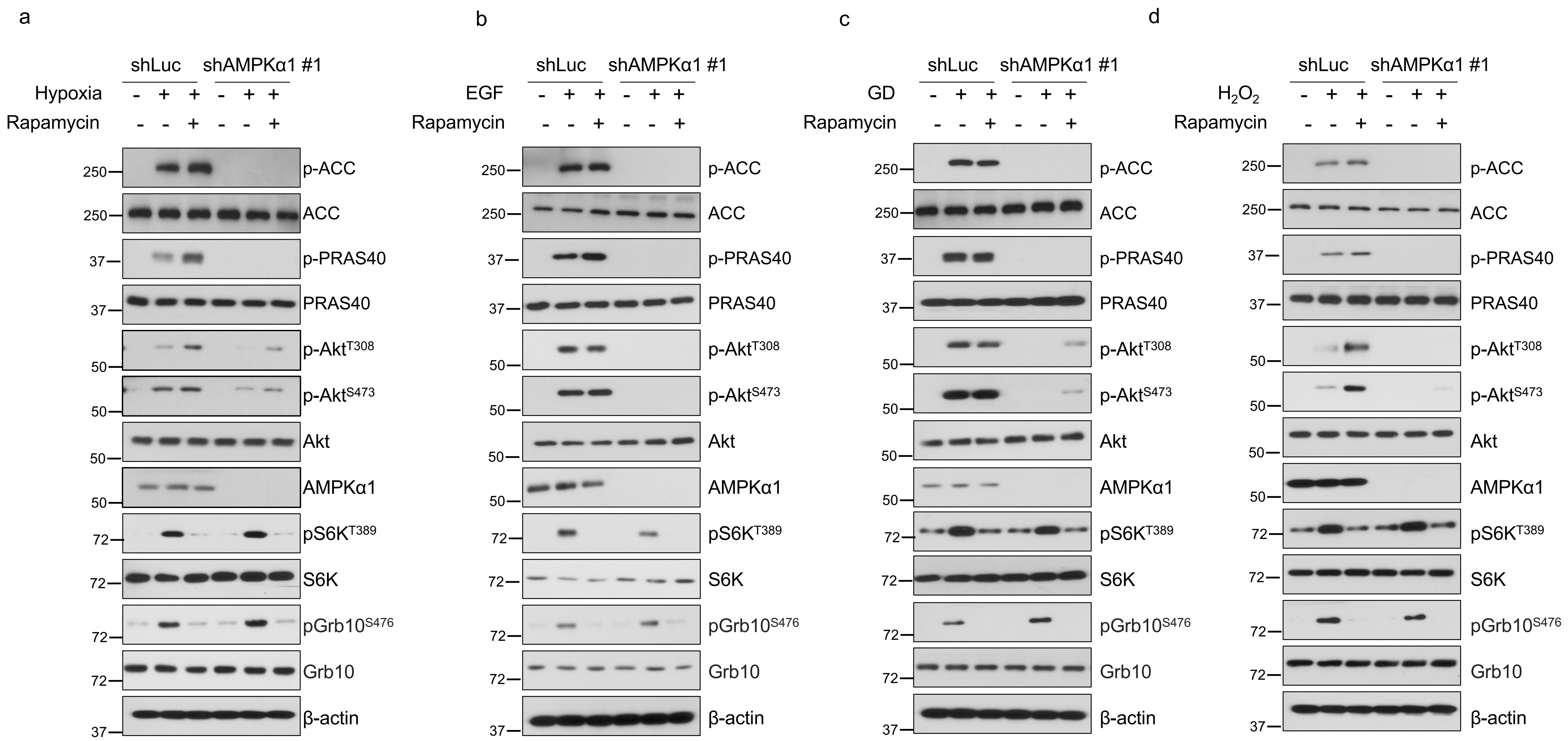

Supplementary Figure 3

Supplementary Fig. 3. AMPK-mediated Skp2 S256 phosphorylation and Akt activation is mTOR independent.

(a) Immunoblotting for control (shLuc) and AMPKα1 knockdown (#1) MDA-MB-231 cells pretreated treated with or without Rapamycin (10 μM) and challenged with hypoxia (1% O2) for 4 hours. **(b)** Immunoblotting of control (shLuc) and AMPKα1 knockdown (#1) MDA-MB-231 cells serum starved and treated with EGF for 5 minutes. **(c)** Immunoblotting of with control (shLuc) and AMPKα1 knockdown (#1) MDA-MB-231 cells under glucose deprivation for 4 hours. **(d)** Immunoblotting of with control (shLuc) and AMPKα1 knockdown (#1) MDA-MB-231 cells under H_2O_2 (100 μM) for 2 hours.

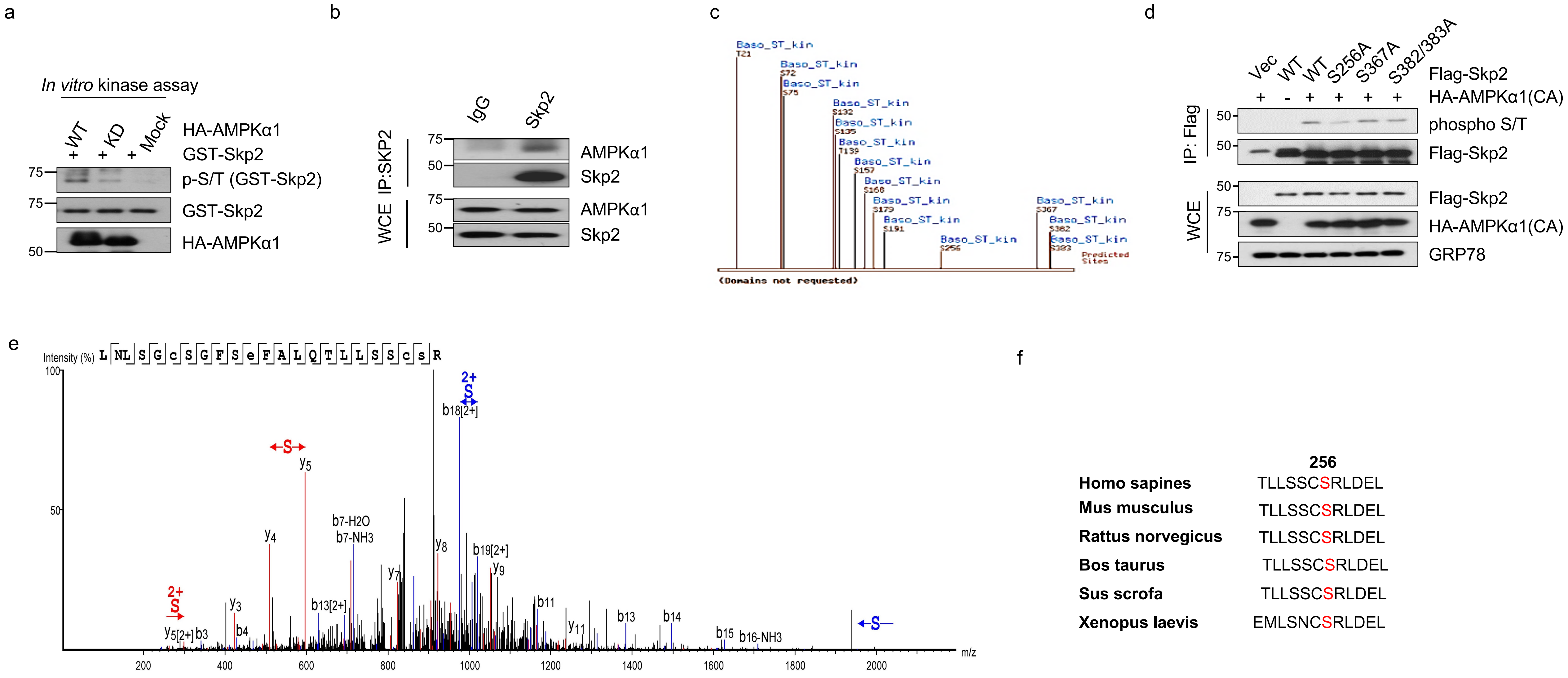

Supplementary Figure 4

Supplementary Fig. 4. AMPK phosphorylates Skp2 at S256 and maintains Skp2 SCF complex integrity.

(a) *In vitro kinase assay* of GST-Skp2 and HA-AMPK. **(b)** HEK293T cells were immunoprecipitation with Skp2 antibody, followed by immunoblotting. **(c)** Skp2 putative phosphorylation sites by AMPK are revealed by Scansite (http://scansite.mit.edu/). Scansite was used on the lowest stringency settings. **(d)** HEK293 cells transfected with Skp2 WT, S256A, S367A, S382A/S383A and the indicated HA-AMPKα (CA) constructs were subjected to immunoprecipitation, followed by immunoblotting. **(e)** HEK293 cells were transfected with or without HA-AMPKα (CA). Cell lysate with phosphatase inhibitors were subject to MS/MS analysis. **(f)** The sequence of Skp2 in different species are listed. The conserved Serine (S) residues at 256 in different species are highlighted.

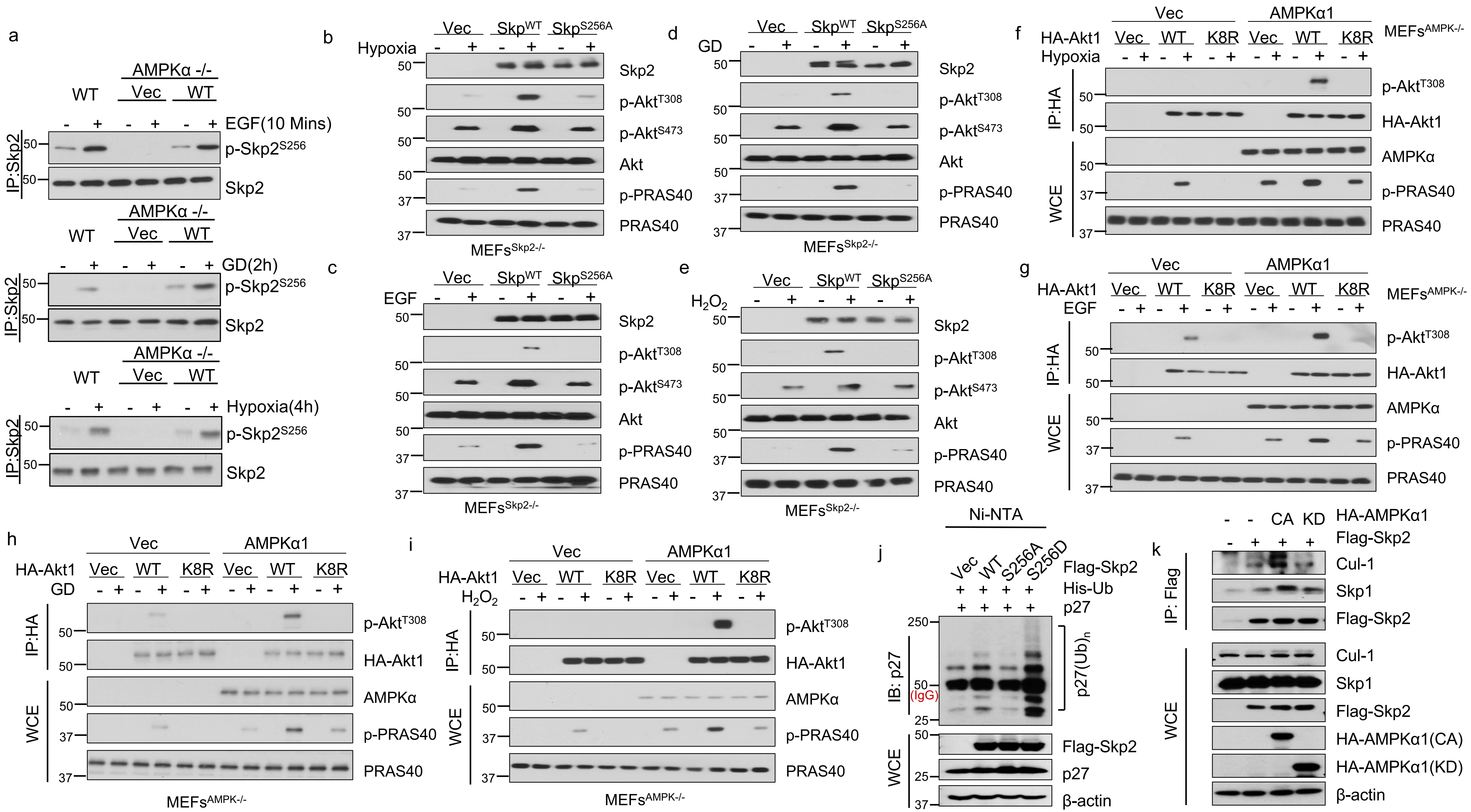

Supplementary Figure 5

Supplementary Fig. 5. AMPK regulates Akt signaling under EGF and stress through Skp2 S256 phosphorylation.

(**a**) Immunoblotting of WT and AMPK null MEFs and WT AMPK addback under EGF, glucose deprivation and hypoxia. (**b-e**) Immunoblotting SKP2 null MEFs restored with SKP2 WT, SKP2 S256A under hypoxia (1% O_2) for 4 hours, EGF (50 ng/ml) for 10 mins, glucose deprivation for 2 hours and H_2O_2 for 1 hour. (**f-i**) Immunoblotting AMPKα null MEFs restored with AMPK, Akt WT and K8R under hypoxia (1% O_2) for 4 hours, EGF (50 ng/ml) for 10 mins, glucose deprivation for 2 hours and H_2O_2 for 1 hour, for each restoration experiment, 5 μg AMPKα1 plasmid and 5 μg HA-Akt wt or HA-Akt K8R mutant plasmid were transfected into indicated cell lines. (**j**) In vivo ubiquitination assay from 293T cells transfected with the indicated plasmids was performed, followed by immunoblotting. **(k)** 293T cells transfected with Flag-Skp2 and the indicated HA-AMPKα (CA/KD) constructs were subjected to immunoprecipitation with Flag antibody, followed by immunoblotting.

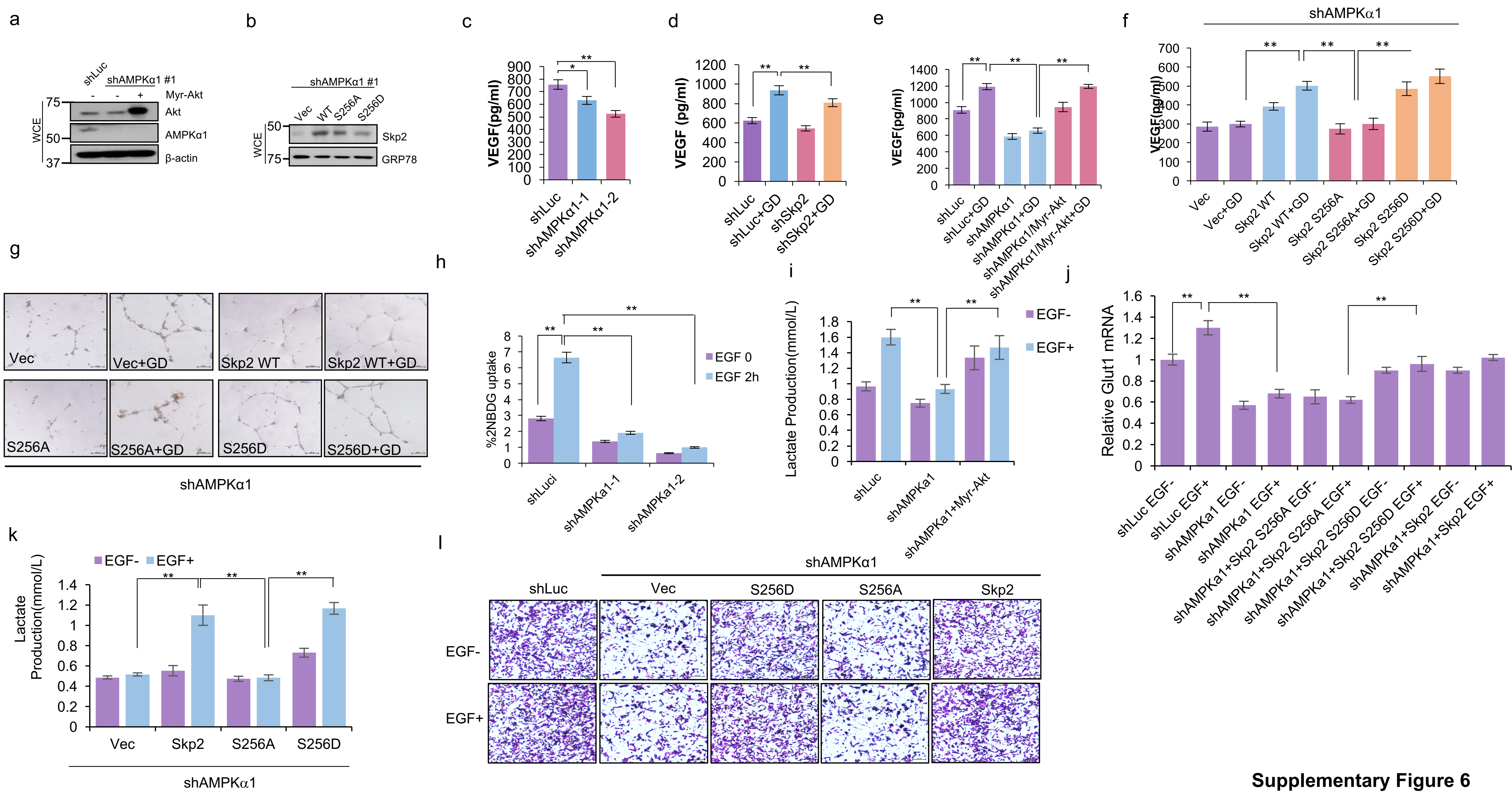

Supplementary Figure 6

Supplementary Fig. 6. AMPK-SKP2-Akt regulates glucose deprivation-induced VEGF production and EGF-induced glycolysis and migration.

(a) Immunoblotting of MDA-MB-231 cells with control (shLuc), AMPKα1 knockdown, AMPKα1 knockdown along with Myr-Akt. **(b)** Immunoblotting of MDA-MB-231 AMPKα1 knockdown cells with Skp2 WT, or Skp2 S256A or Skp2 S256D restoration. **(c-f)** ELISA analysis of VEGF secretion in MDA-MB-231 cells with control (shLuc), AMPKα1 knockdown and AMPKα1 knockdown with Myr-Akt, Skp2 WT, Skp2 S256A or Skp2 S256D restoration with or without glucose for 6 hours. **(g)** Presented image of HUVEC tube formation assay of supernatant from MDA-MB-231 AMPKα1 knockdown with vector control, Skp2 WT and S256A or Skp2 S256D restoration with or without glucose. **(h)** MDA-MB-231 cells with control (shLuc) and AMPKα1 knockdown were starved in DMEM glucose-free medium for 4 hours and added with 2NBDG for 30 minutes. Cells were then subjected to FACS analysis. Each value represents the mean ± SEM (n = 4 per group) in three independent experiments. **P<0.01. **(i)** Lactate production in MDA-MB231 cells with control (shLuc), AMPKα1 knockdown and AMPKα1 knockdown along with Myr-Akt treated with or without EGF (100 ng/ml) for 16 hours. **(j)** RT-PCR analysis of Glut1 transcription in MDA-MB-231 control (shLuc), AMPKα1 knockdown with Vector, Skp2 WT, Skp2 S256A or Skp2 S256D restoration cells with or without EGF (100 ng/ml) for 2 hours. **(k)** Lactate production in MDA-MB-231 AMPKα1 knockdown with Vector, Skp2 WT, Skp2 S256A or Skp2 S256D restoration with or without EGF (100 ng/ml) for 16 hours. **(l)** *In vitro* migration assay in MDA-MB-231 cells with control (shLuc), AMPKα1 knockdown and AMPKα1 knockdown along with Myr-Akt, Skp2 WT, Skp2 S256A or Skp2 S256D restoration treated with or without EGF (50 ng/ml) for 6 hours. Scale bar: 100 μm.

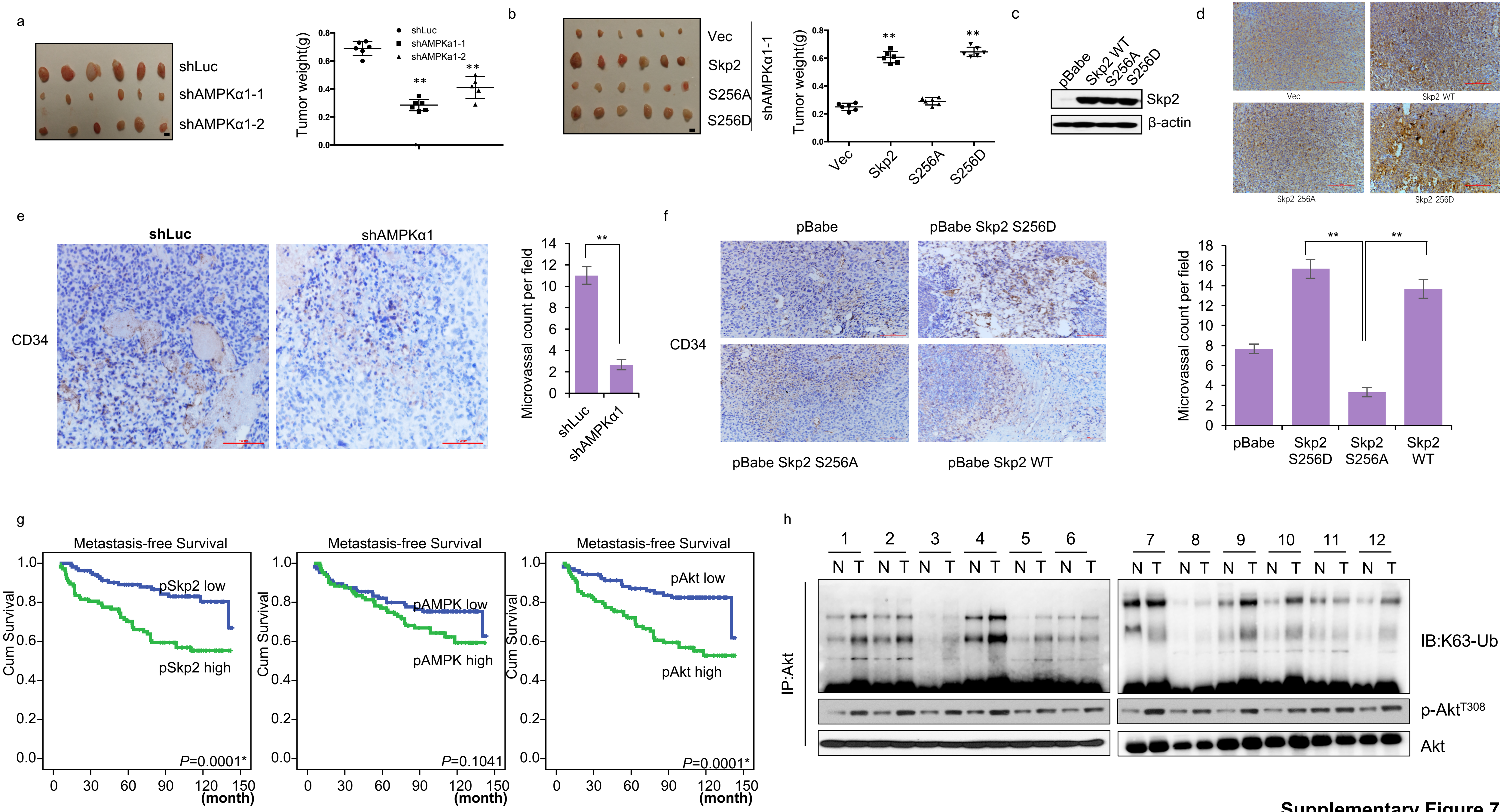

Supplementary Figure 7

Supplementary Fig. 7. AMPK-mediated Skp2 S256 phosphorylation and Akt activation promotes breast cancer progression.

(a, b) MDA-MB-231 cells with control (shLuc) or AMPK knockdown (shAMPKα1) and shAMPKα1-1 were stably transfected with control (Vec), Skp2 WT, Skp2 S256A and Skp2 S256D and subcutaneously injected into nude mice. Tumors are isolated and weighted at Week 4. (n = 6); **, P <0.01. **(c)** Immunoblotting of MDA-MB-231 cells with stable expression of the indicated pBabe vector, Skp2 WT, S256A and S256D. **(d)** IHC staining of Akt and phosphor-Akt T308 in parafilm embedded section of subcutaneous tumors from AMPK knockdown MDA-MB-231 cells restored with Vector, Skp2 WT, S256A or S256D mutant. **(e, f)** CD34 IHC staining of mouse xenograft from MDA-MB-231 control (shLuc), AMPKα1 knockdown and MDA-MB-231 overexpression of pBabe vector, Skp2 WT, S256A and S256D. Micro-vassal were counted from randomly field; Columns, mean (n = 3); bars, mean ± S.D. **, P <0.01. **(g)** Overexpression of pSkp2 and pAkt predicts poor survival outcome of breast cancer patients. Kaplan-Meier plots showed that high expression of pAMPK (Left panel), pSkp2 (S256) (Middle panel) and pAkt (Right panel) significantly predicted metastasis-free survival. p-values were shown in the graphs. **(h)** Frozen samples from triple negative breast cancer patients were lysed and immunoprecipitation with Akt antibody, followed by immunoblotting.

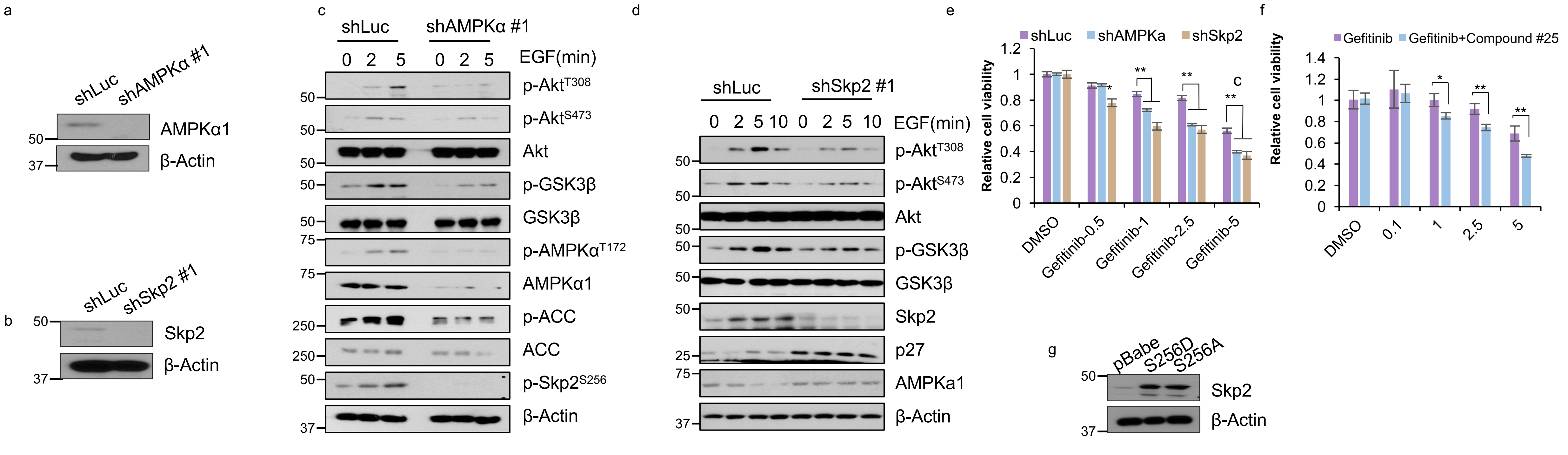

Supplementary Figure 8

Supplementary Fig. 8. AMPK-mediated Skp2 S256 phosphorylation and Akt activation contributes to resistance of EGFR targeting therapy.

(a, b) Immunoblotting of AMPKa1 and Skp2 knockdown efficiency in H1975 Gefitinib-resistant cell line. **(c, d)** Immunoblotting of AMPKa1 and Skp2 knockdown in H1975 Gefitinib-resistant cell line treated with EGF (50 ng/ml) for indicated time. **(e)** AMPK and Skp2 knockdown Gefitinib-resistant H1975 NSCLC cells were treated with Gefitinib at the indicated concentrations for 72 h, and cell viability was analyzed by cell counting kit 8. Columns, mean (n = 3); bars, mean ± S.D. *, $P < 0.05$; **, $P<0.01$. **(f)** Cell viability analysis of Gefitinib-resistant H1975 NSCLC cells treated with compound #25 together with indicated concentrations of Gefitinib for 72 hours. Columns, mean (n = 3); bars, mean ± S.D. *, $P < 0.05$; **, $P<0.01$. **(g)** Immunoblotting of H1975 cells with stable overexpression of pBabe vector control, pBabe-Skp2 S256A or pBabe-Skp2 S256D.

a

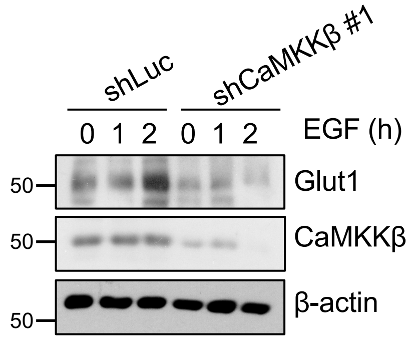

b

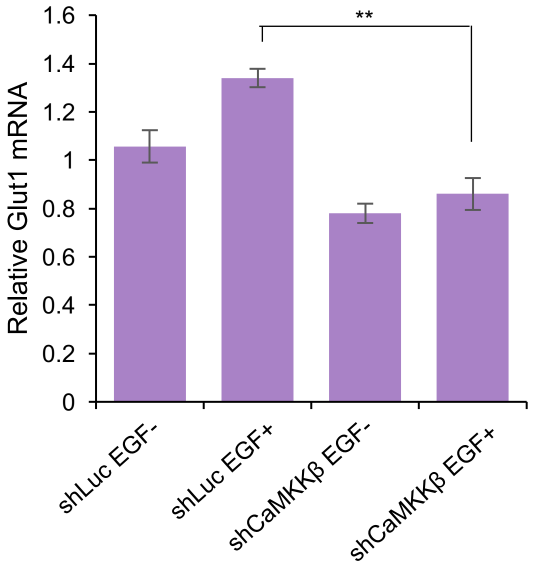

Supplementary Figure 9

Supplementary Fig. 9. EGF-induced Glut1 expression was impaired in CaMKKβ knockdown cells.

(a) Immunoblotting of control (shLuc) and CaMKKβ knockdown MDA-MB-231 cells serum starved and treated with EGF for 1 and 2 hours. **(b)** RT-PCR analysis of Glut1 transcription in control (shLuc) and CaMKKβ knockdown MDA-MB-231 cells with and without EGF for 2 hours.

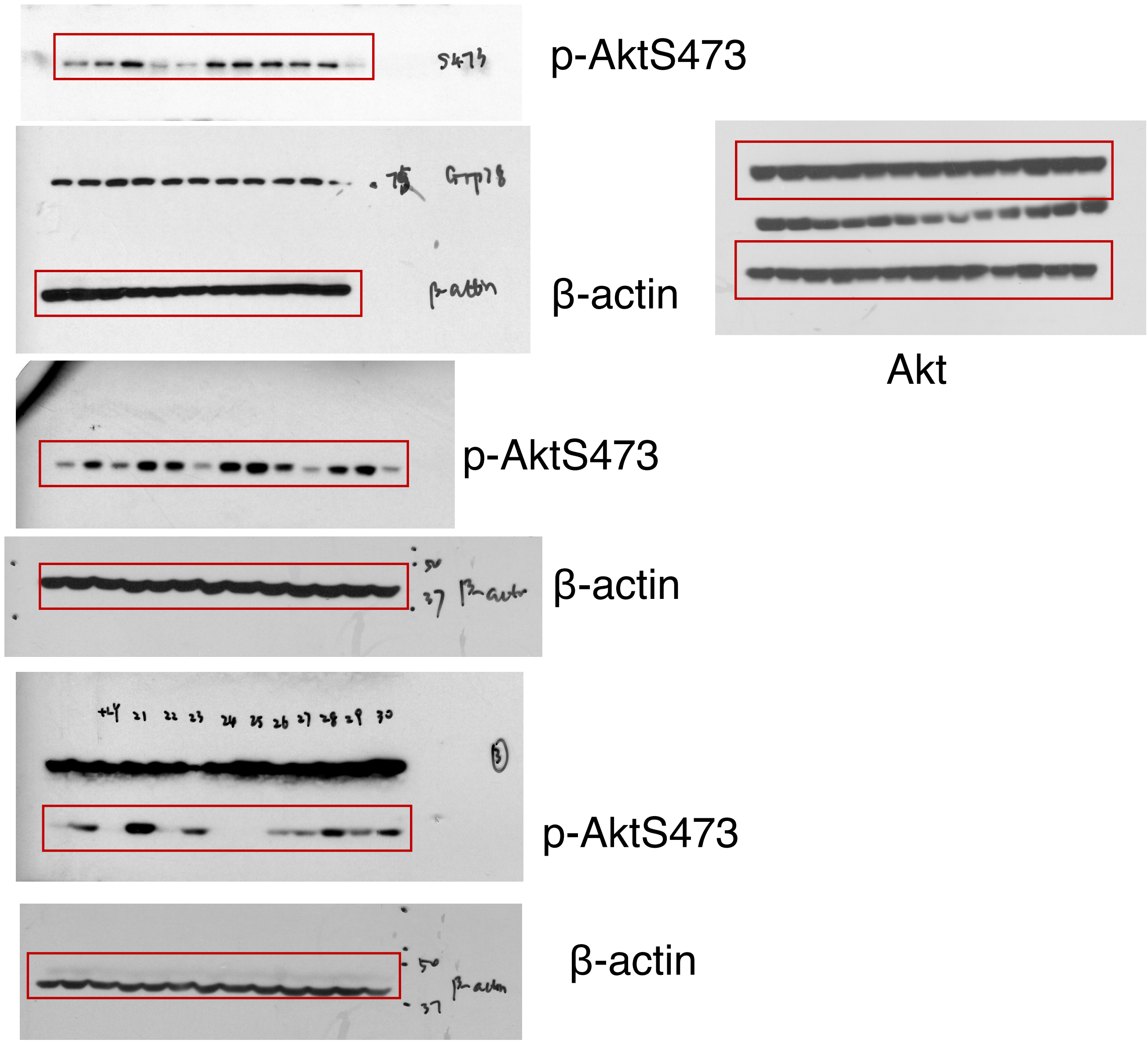

Figure 1 a

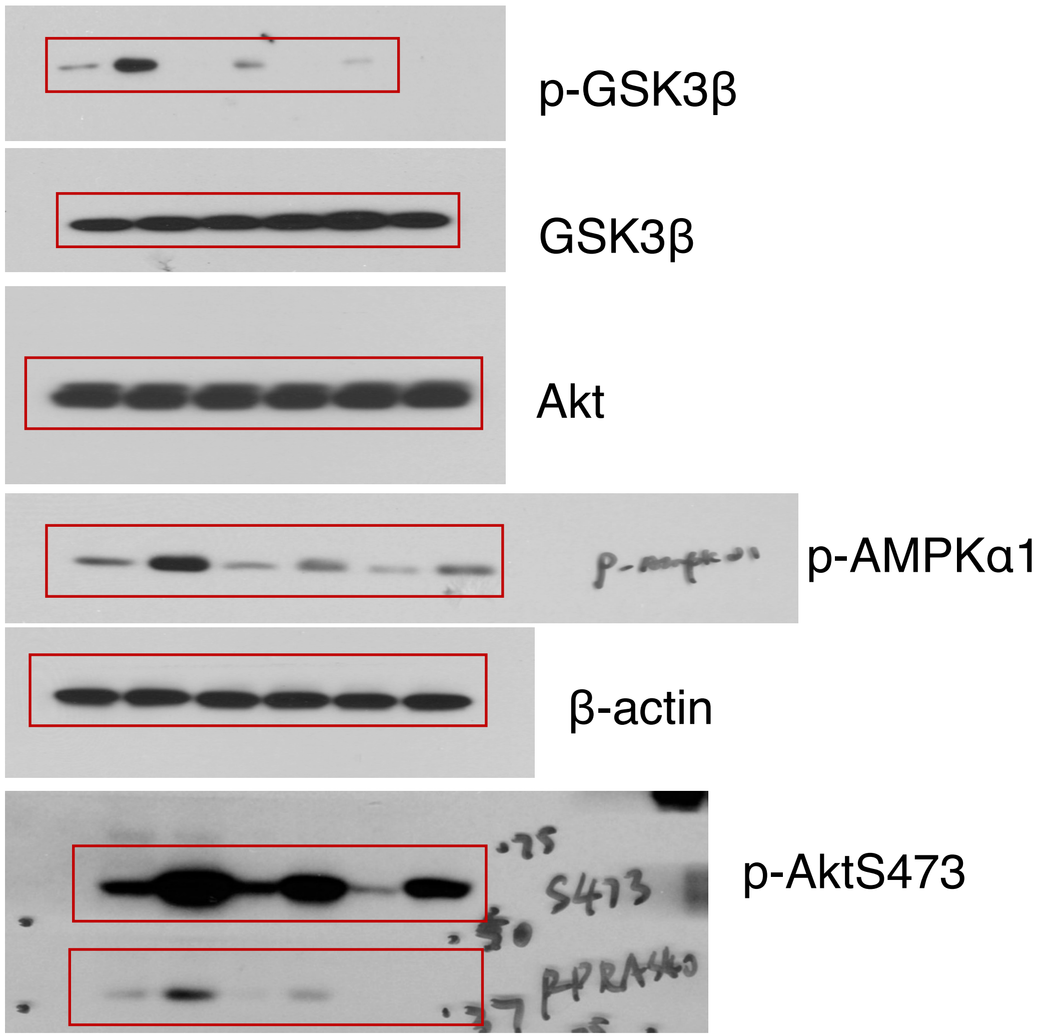

Figure 1 b

Supplementary Fig. 10 Uncropped original blots for Figure 1a and Figure 1b

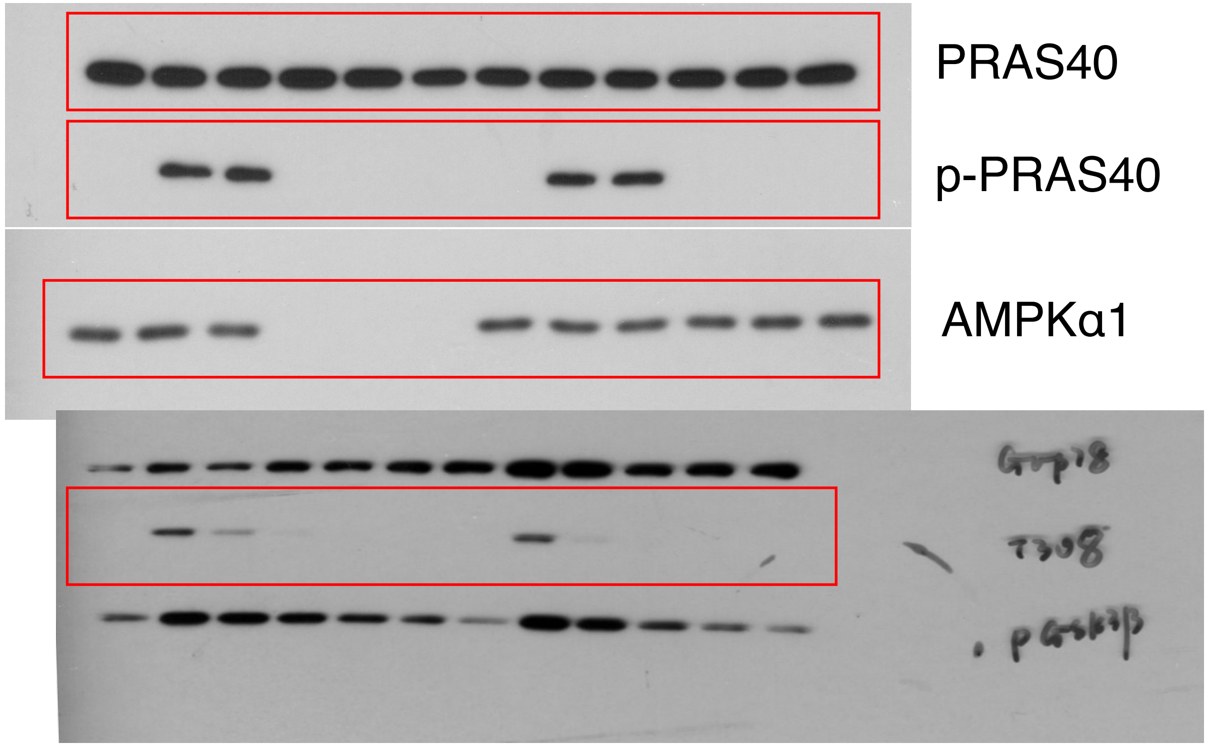

Figure 1 c

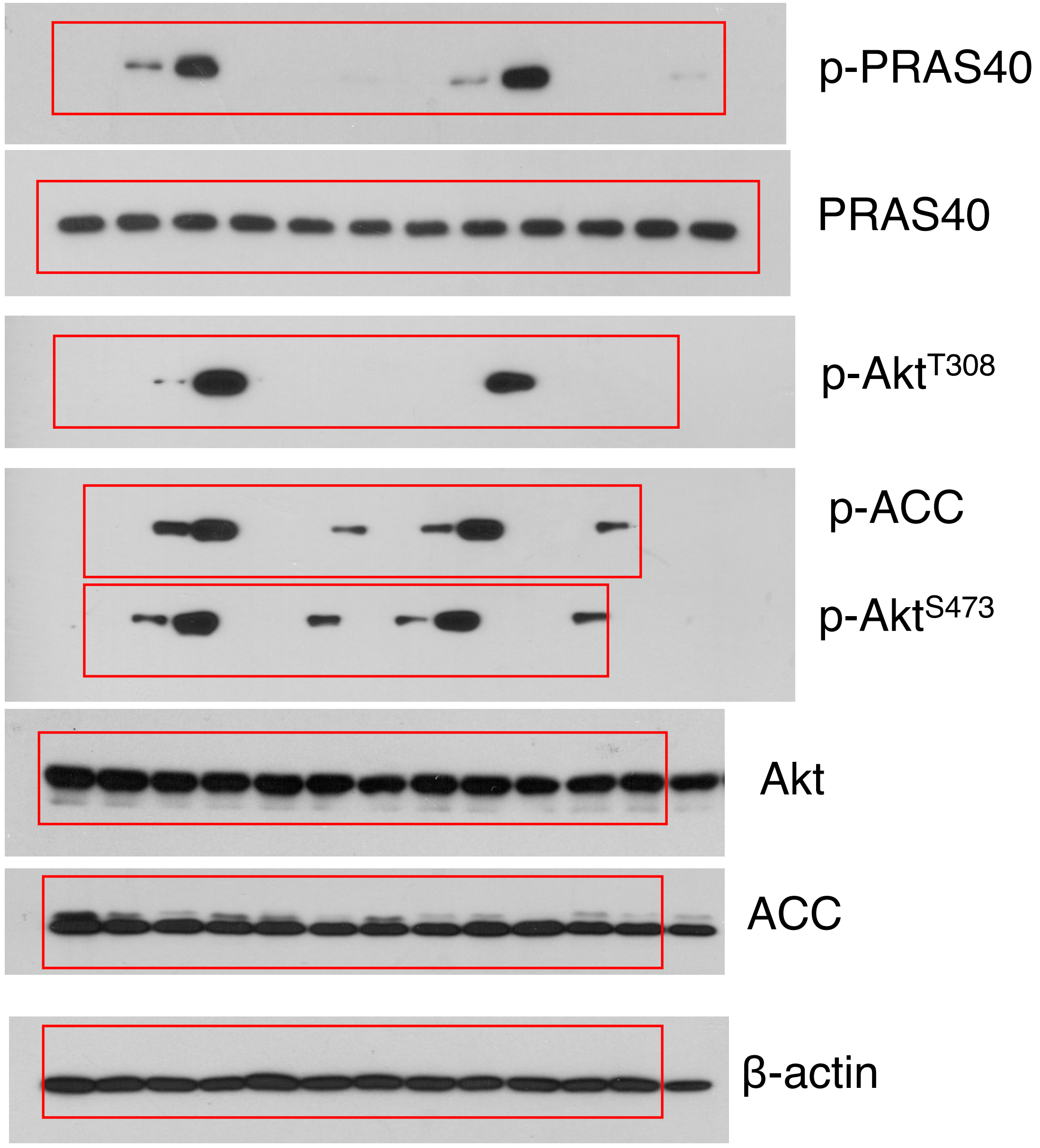

Figure 1 d

Supplementary Fig. 11 Uncropped original blots for Figure 1c and Figure 1d

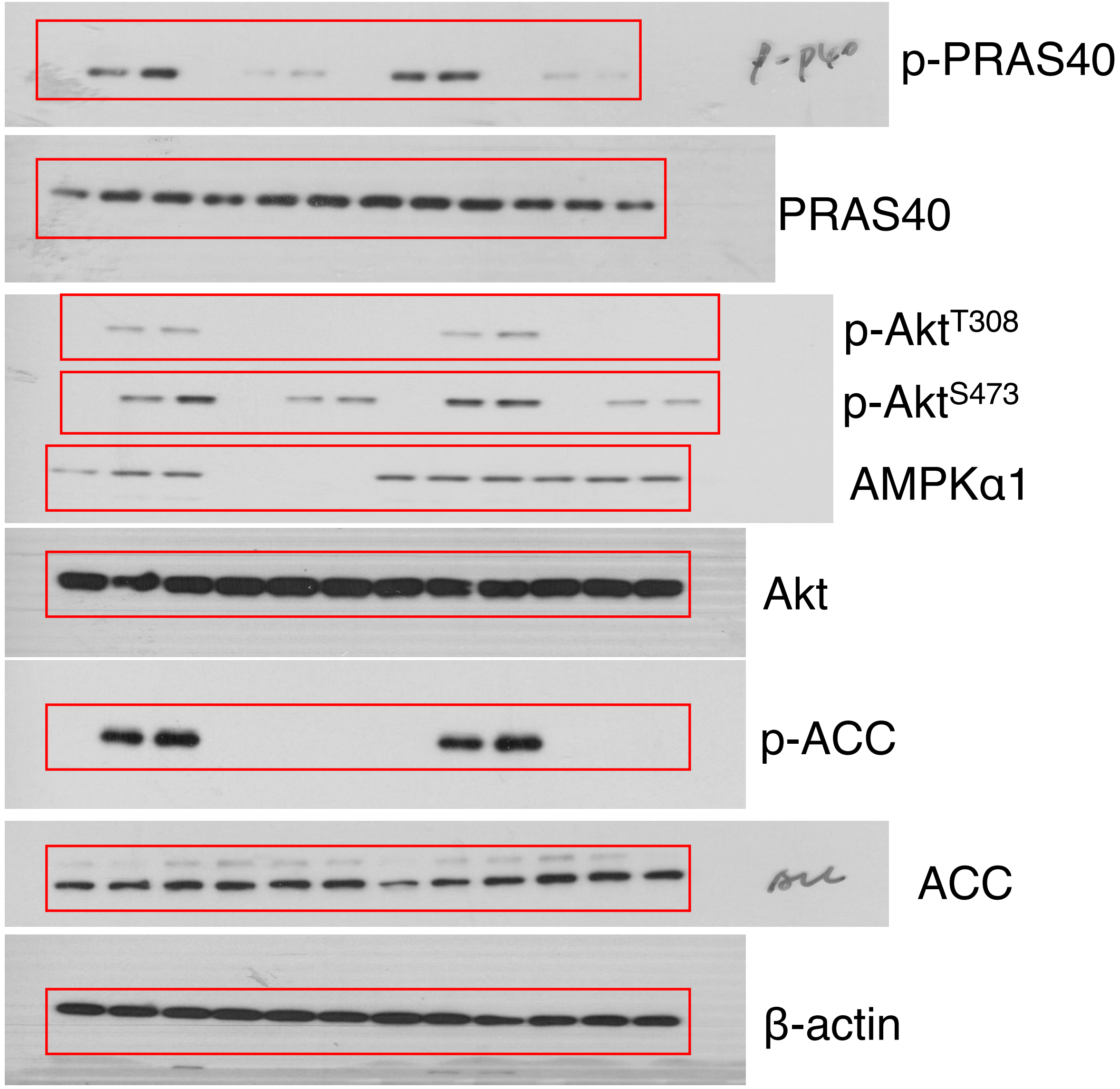

Figure 1 e

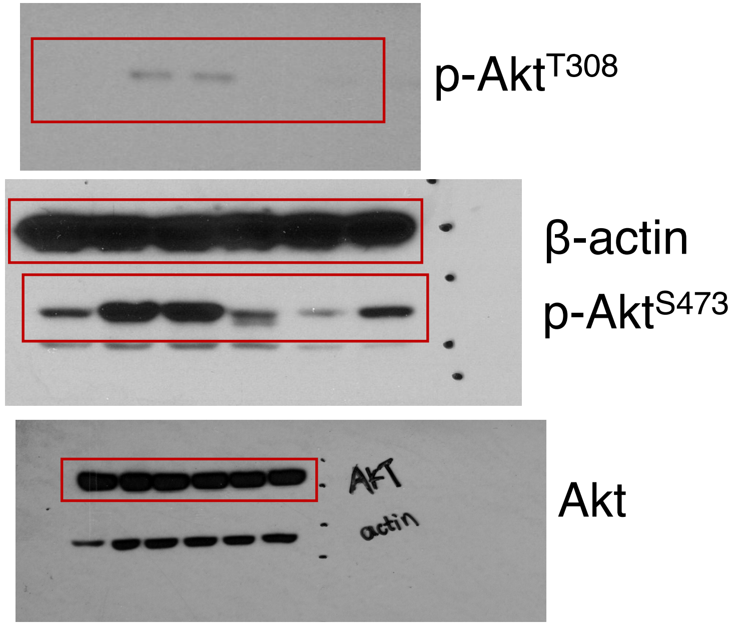

Figure 1 f

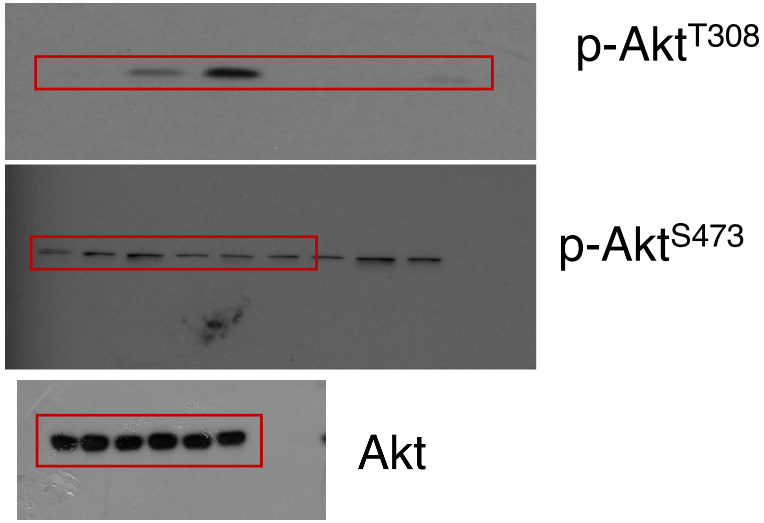

Figure 1 g

Supplementary Fig. 12 Uncropped original blots for Figure 1e-1g

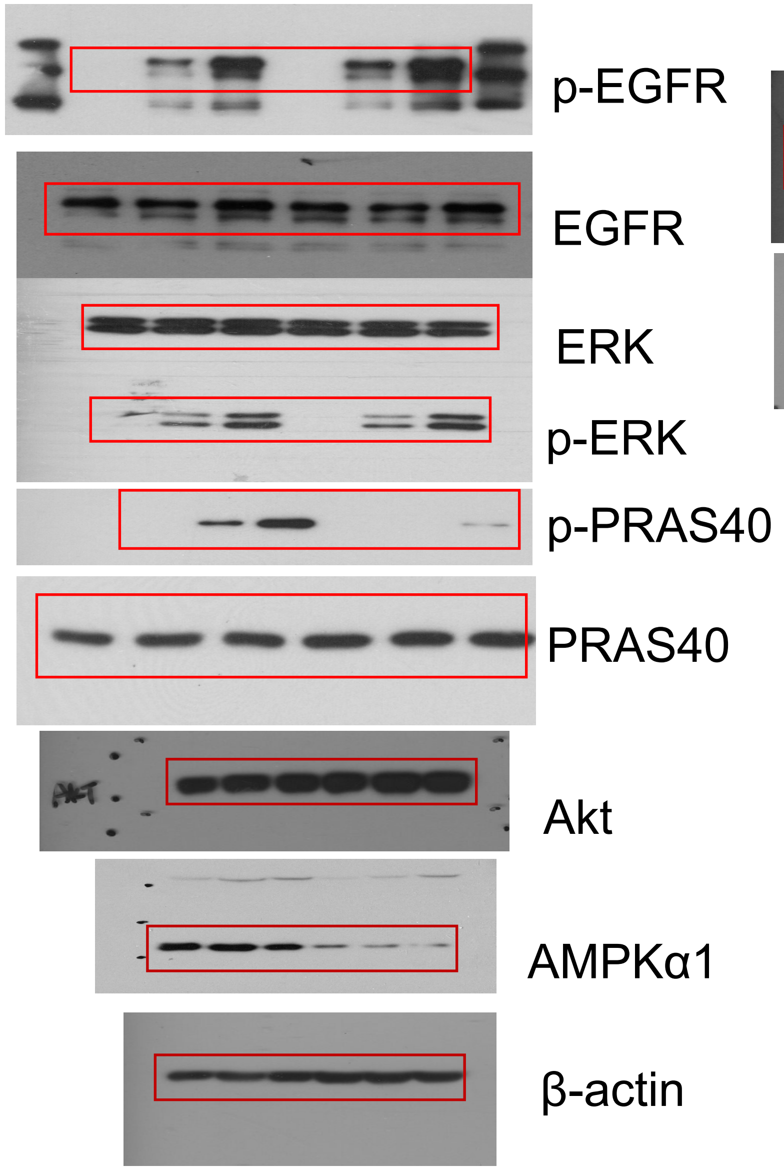

Figure 2 a

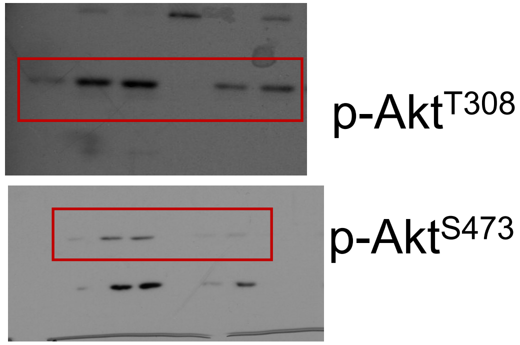

Figure 2 a

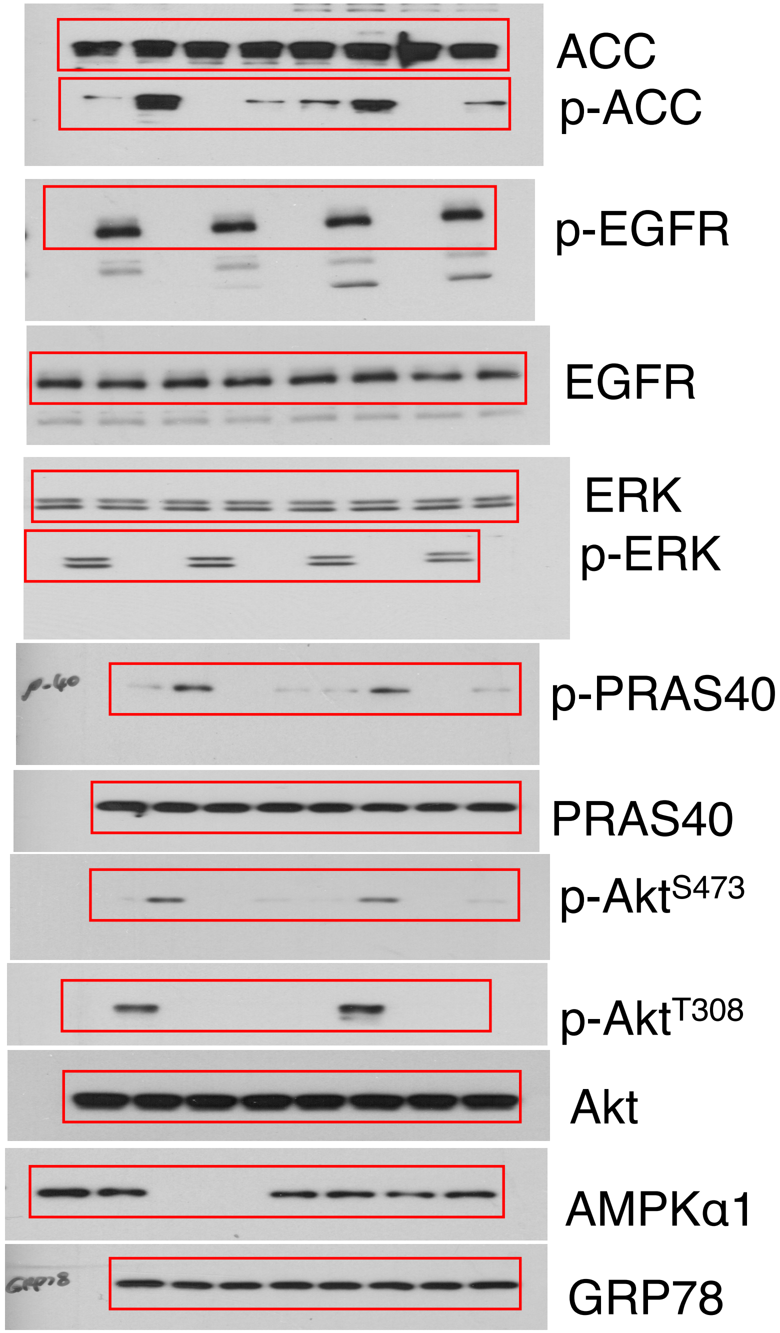

Figure 2b

Supplementary Fig. 13 Uncropped original blots for Figure 2a and 2b

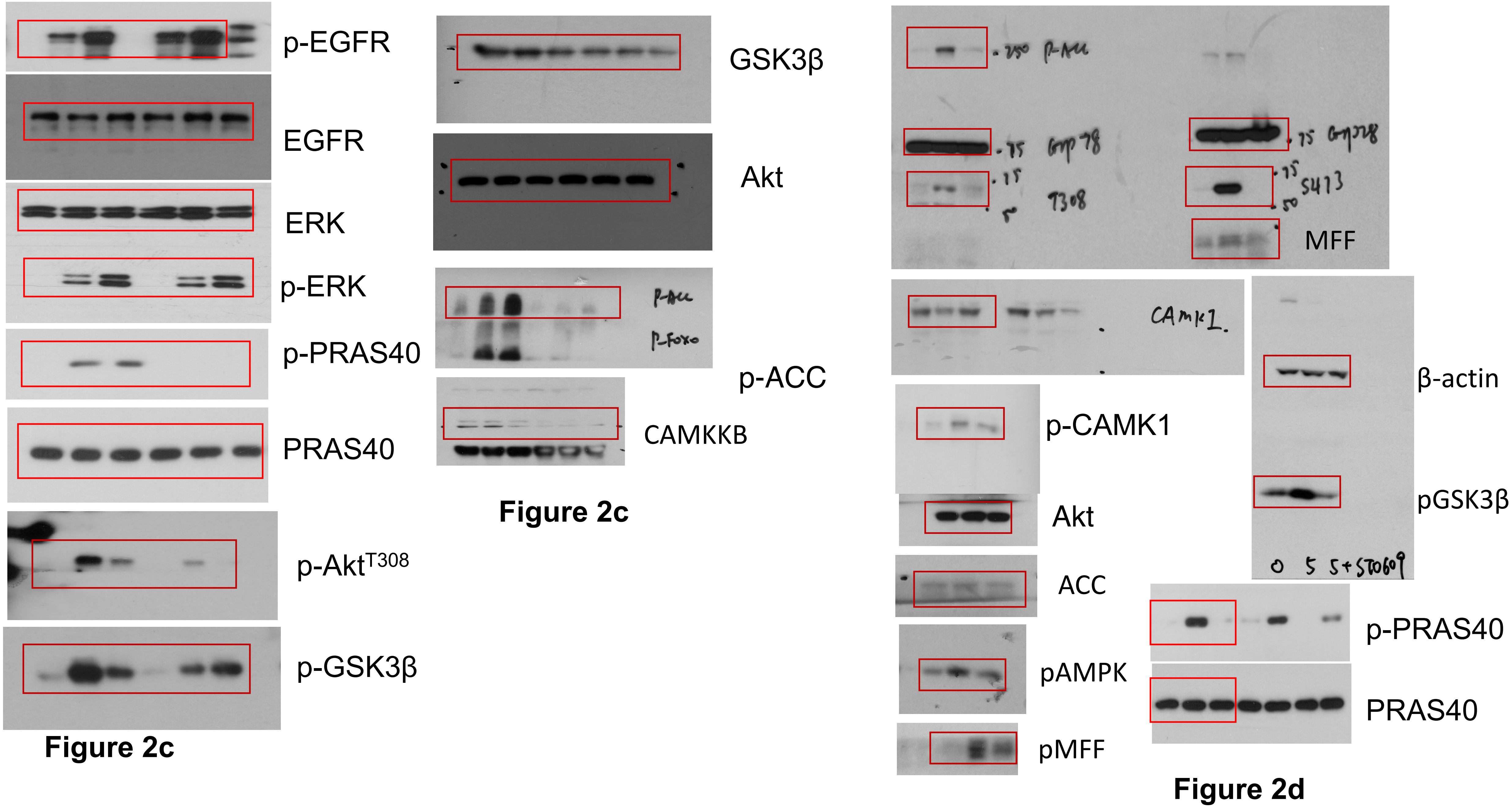

Supplementary Fig. 14 Uncropped original blots for Figure 2c and 2d

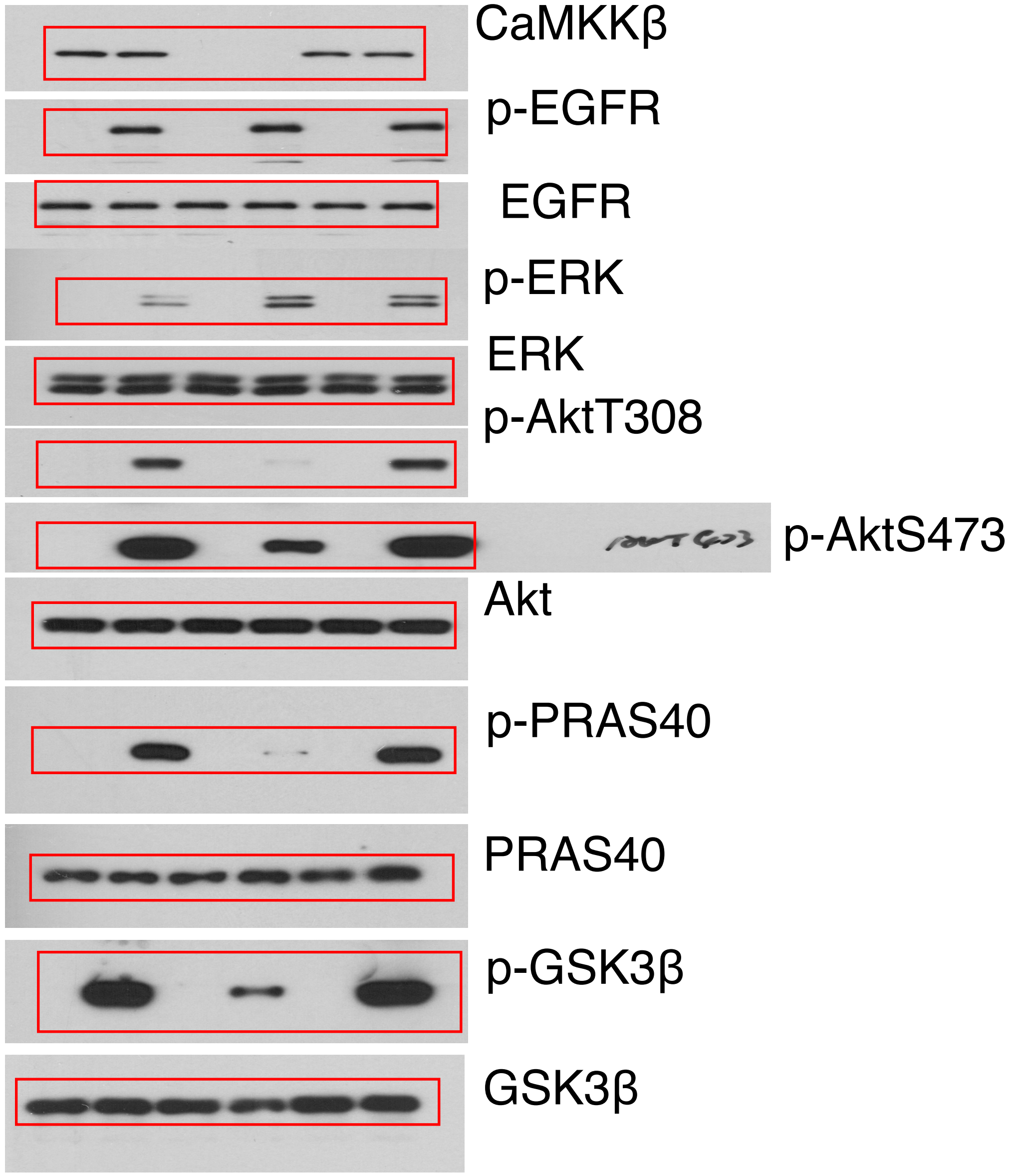

Figure 2e

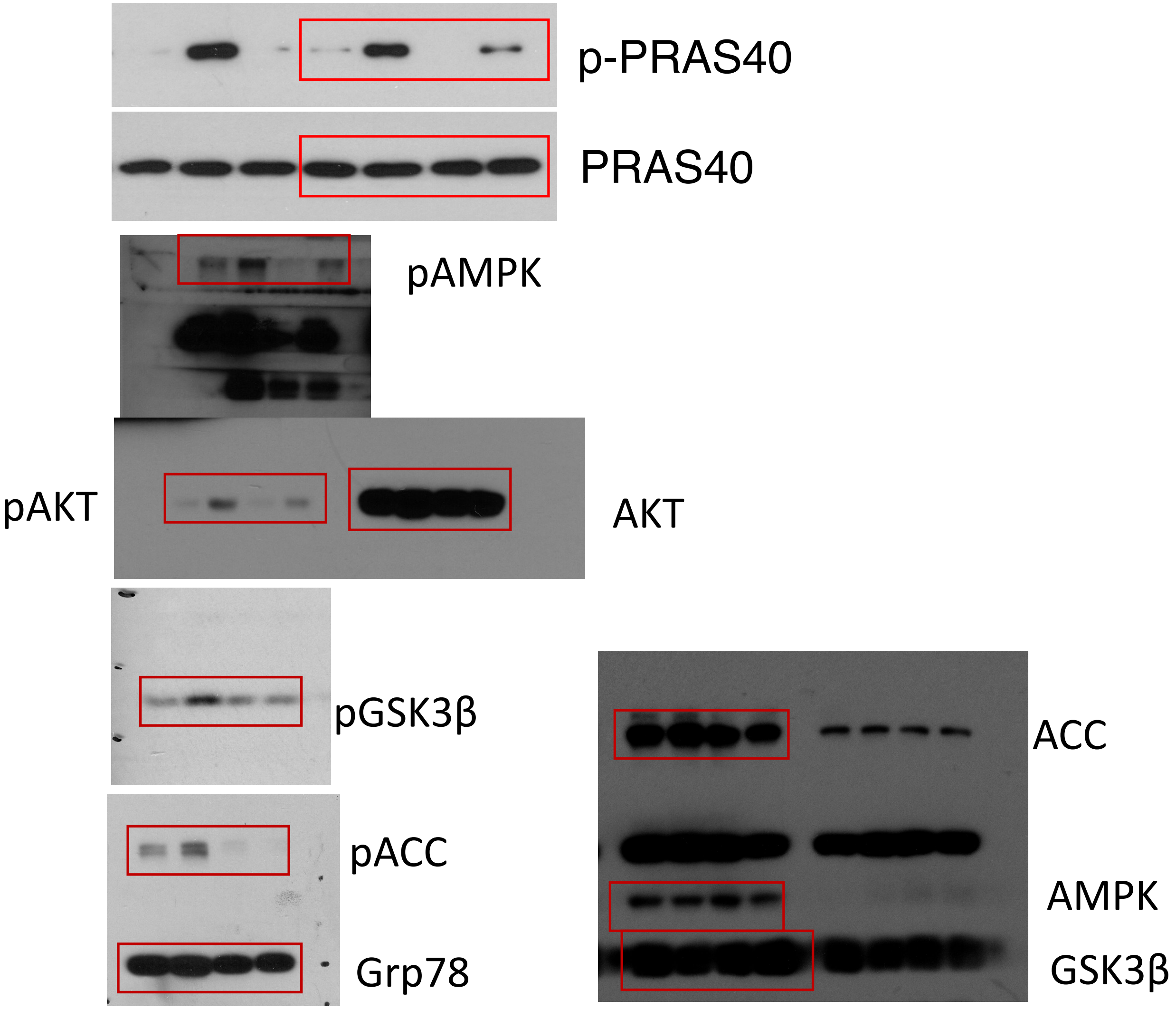

Figure 2f

Supplementary Fig. 15 Uncropped original blots for Figure 2e and 2f

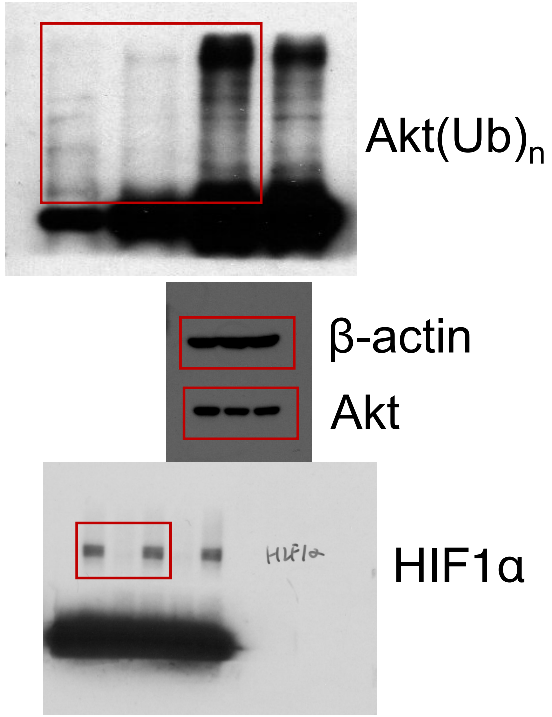

Figure 3a

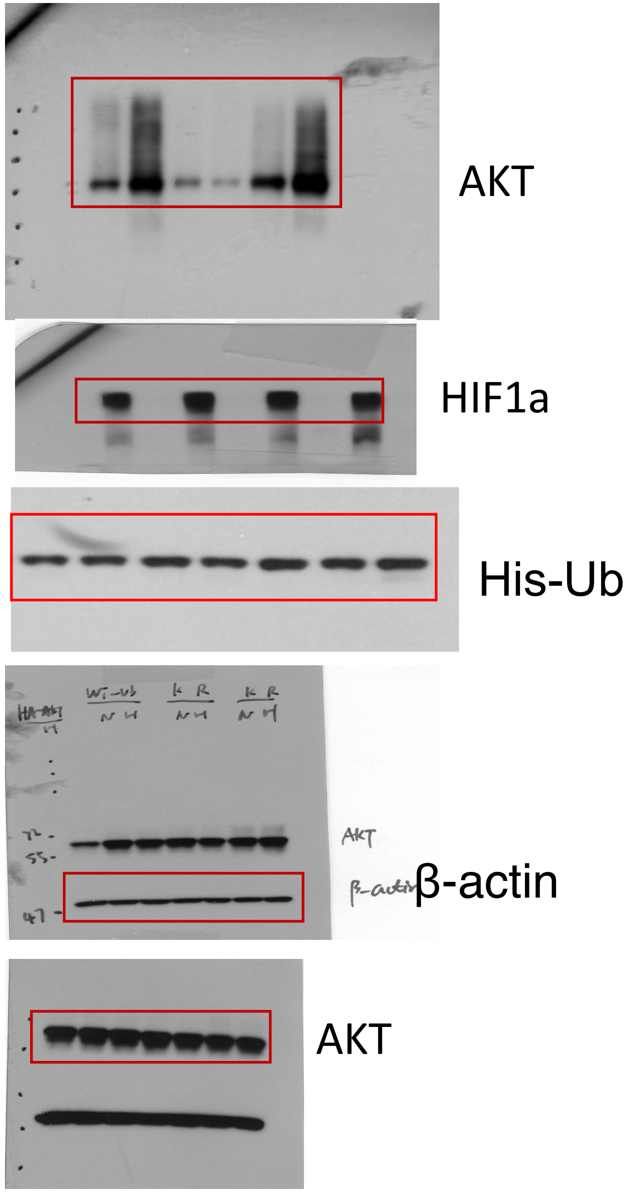

Figure 3b

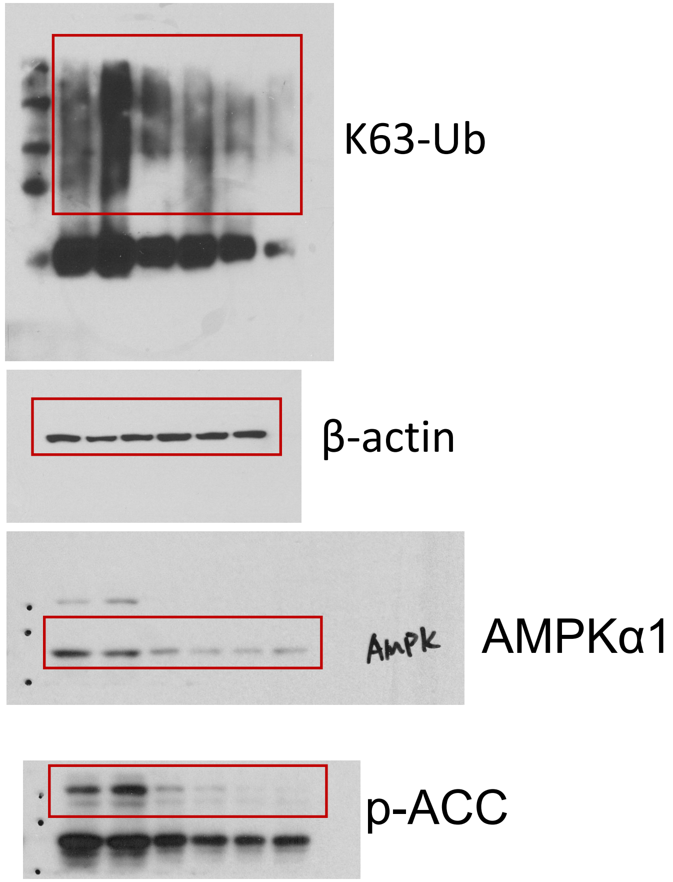

Figure 3c

Supplementary Fig. 16 Uncropped original blots for Figure 3a-3c

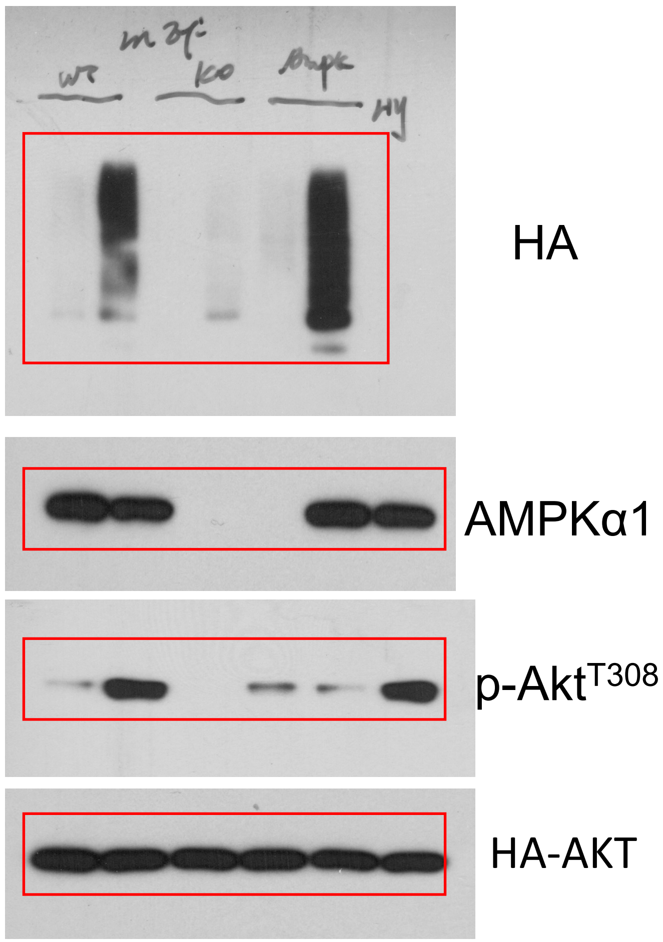

Figure 3d

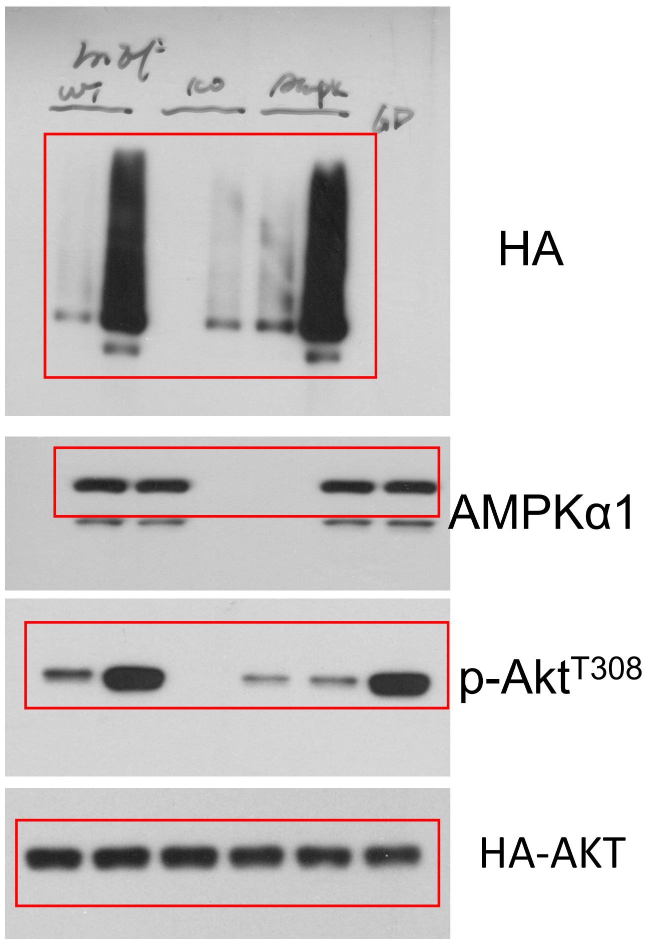

Figure 3e

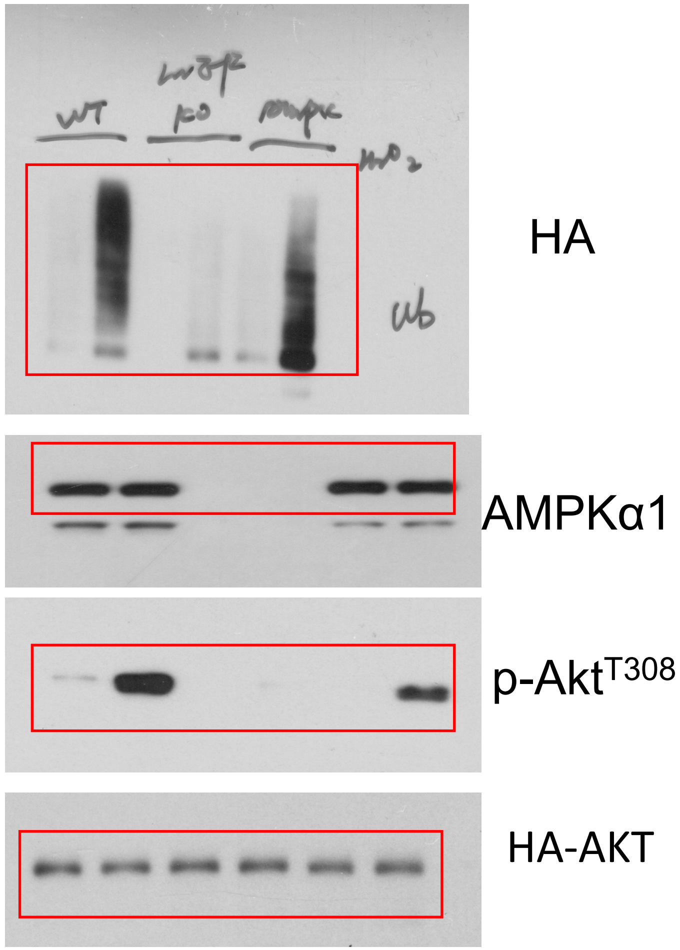

Figure 3f

Supplementary Fig. 17 Uncropped original blots for Figure 3d-3f

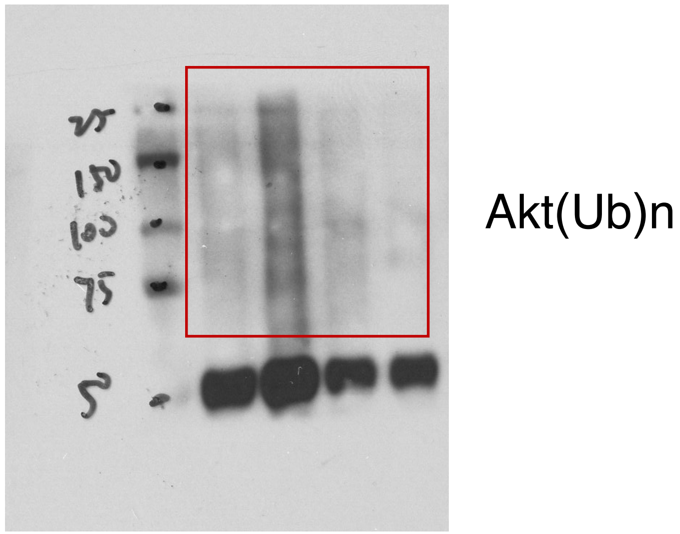

Figure 3g

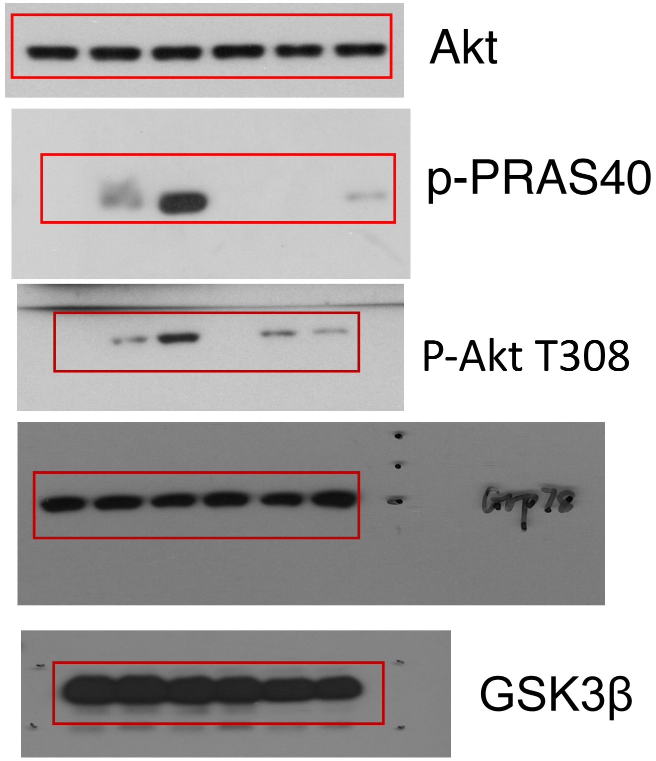

Figure 3h

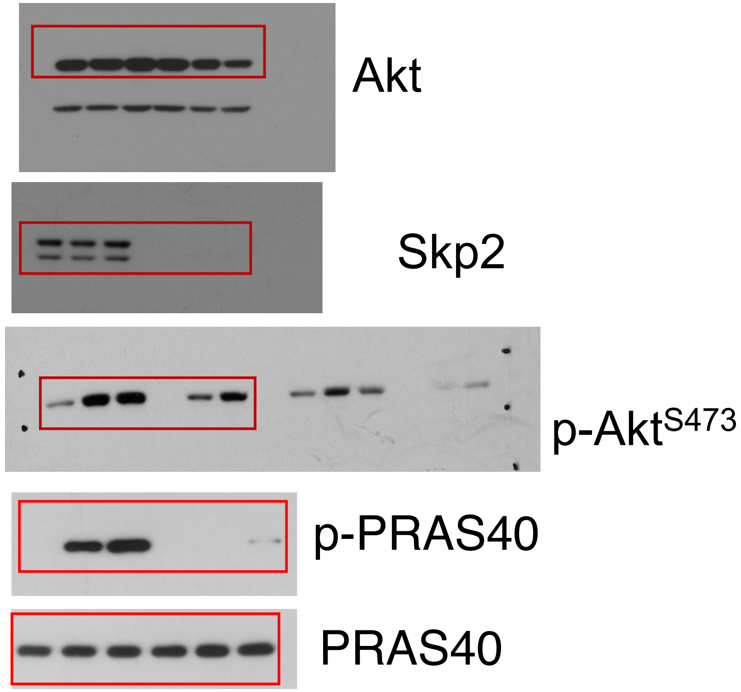

Figure 3i

Supplementary Fig. 18 Uncropped original blots for Figure 3g-3i

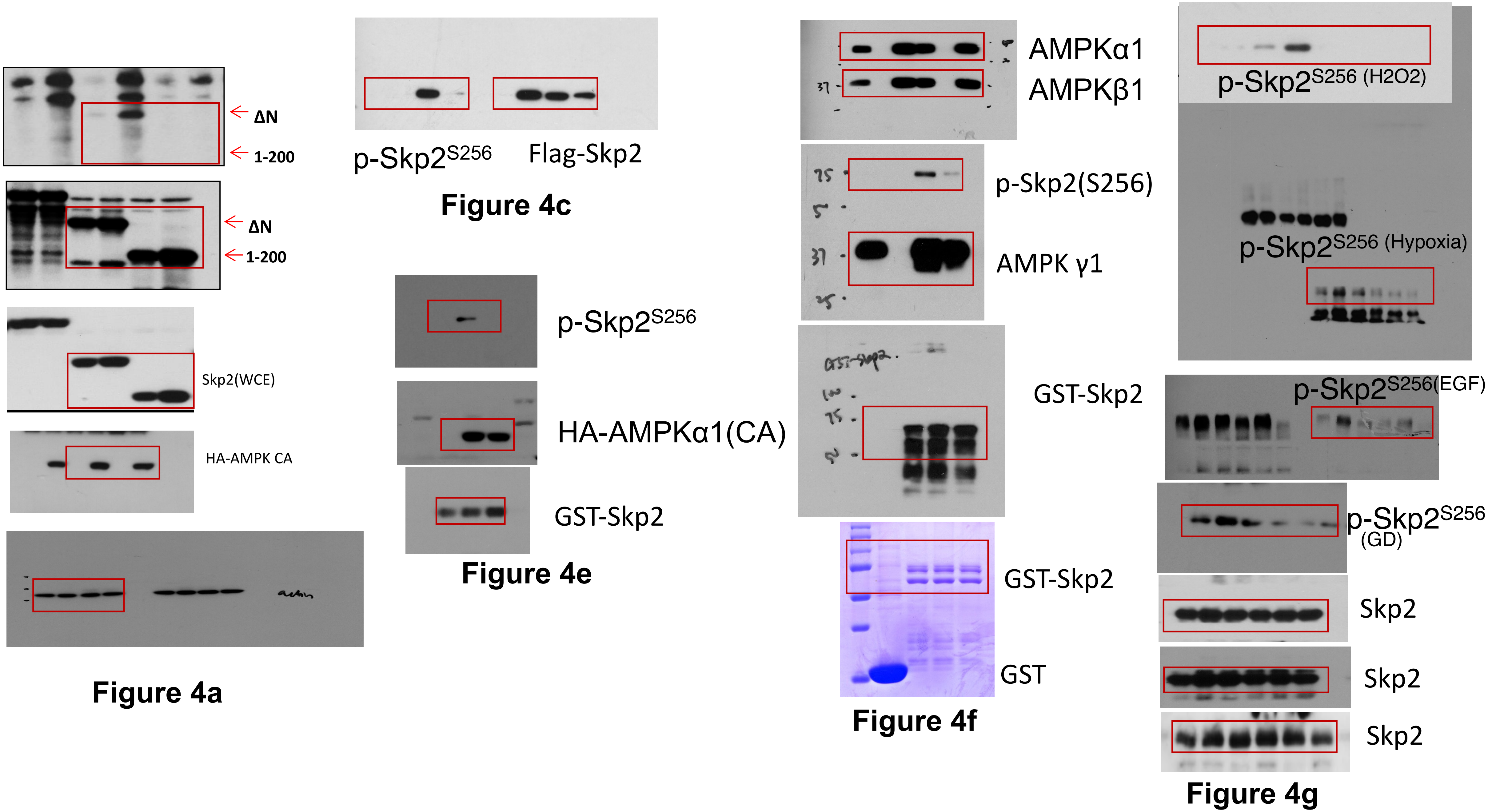

Supplementary Fig. 19 Uncropped original blots for Figure 4a-4g

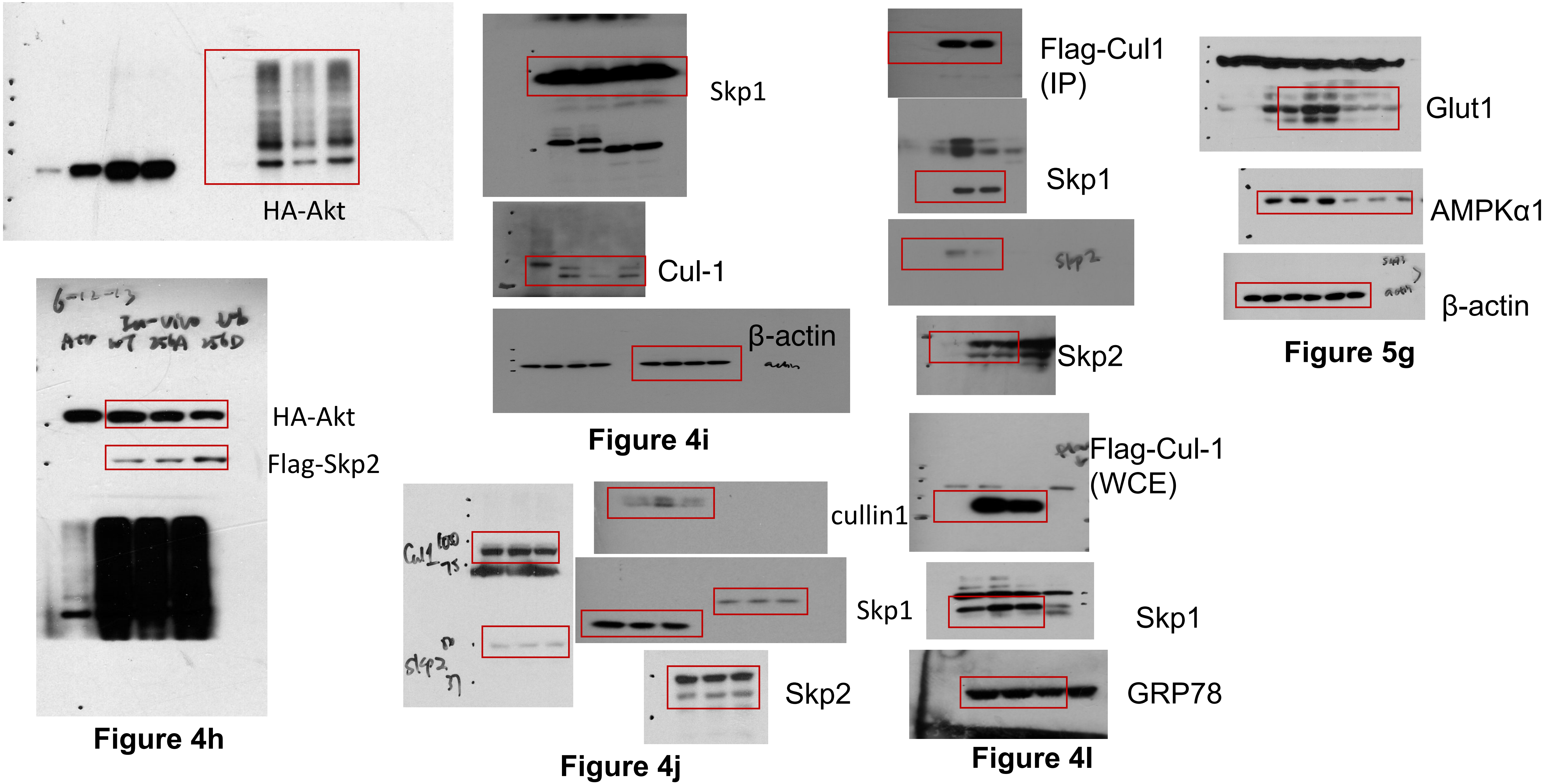

Supplementary Fig. 20 Uncropped original blots for Figure 4h-4l and 5g

Product Name	Targets	Pathway
LY294002	PI3K	PI3K/Akt/mTOR
pp2	Src	Protein tyrosine kinase
Imatinib	BCR-ABL	Protein tyrosine kinase
Nilotinib	BCR-ABL	Protein tyrosine kinase
CX-4945	CK2	DNA Repair
KU-55933	ATM	DNA Repair
KU-57788	DNAPK	DNA Repair
Compound C	AMPK	Metabolism
Quizartinib (AC220)	FLT3	Angiogenesis
GNE-0877	LRRK2	Autophagy
BMS-536924	IGF1R	Protein tyrosine kinase
PF-562271	FAK	Angiogenesis
BI2536	FLK	Cell Cycle
Palbociclib	CDK	Cell Cycle
SGI-1776	PIM	JAK/STAT
ZCL278	RAC	Cell Cycle
SKI II	S1P Receptor	GPCR&G Protein
Crizotinib	c-Met, ALK	Protein tyrosine kinase
CUDC-101	HDAC,HER2, EGFR	Epigenetics
OSU-03012	PDK-1	PI3K/Akt/mTOR
K-Ras(G12C) inhibitor 9	Rho	Cell Cycle
Nintedanib	VEGFR,PDGFR,FGFR	Protein tyrosine kinase
Cabozantinib	FLT3, Tie-2,c-Kit, c-Met,VEGFR	Protein tyrosine kinase
Selumetinib	MEK	MAPK
SB 203580	p38, MAPK	MAPK
H 89 2HCL	PKA	PI3K/Akt/mTOR
TWS119	GAK-3	PI3K/Akt/mTOR
Enzastaurin	PKC	TGF-beta/Smad

Supplementary Table 1

Supplementary Table 1. List of kinase inhibitors and their targeted proteins and pathways.

Table 2 Associations between pSkp2 expressions with important clinic pathologic variables and pAkt, p27, pAMPK expression

Parameter	Category	Case No.	pSkp2 LI		pAkt LI		p27 LI		pAMPK	
			H-Score	p-value	H-Score	p-value	H-Score	p-value	H-Score	p-value
Primary tumor (T)[#]	T1	90	157.1±38.49	0.010*	206.3±52.77	0.053	180.7±64.79	0.395	168.2±61.23	0.759
	T2	96	166.7±47.63		218.3±57.04		189.5±67.18		176.8±69.28	
	T3-T4	22	186.8±46.79		235.7±56.30		196.6±57.78		190.9±80.87	
Nodal status (N)[&]	N0	122	158.9±41.33	0.014*	208.6±52.82	0.106*	186.7±66.03	0.990	172.3±64.98	0.721
	N1-N2	86	172.7±47.70		224.0±58.53		186.1±64.26		177.8±71.02	
Stage[#]	I	69	156.3±37.24	0.004*	202.6±51.37	0.004*	176.5±61.43	0.308	165.6±59.66	0.007*
	II	115	163.0±42.83		214.8±52.48		192.4±69.18		170.4±67.94	
	III	24	196.5±57.79		251.5±67.54		186.7±53.44		220.4±70.73	
pAkt[%]		208	r=0.282	<0.001*	-	-	-	-	-	-
p27[%]		208	r=-0.250	<0.001*	r=0.107	0.125	-	-	-	-
pAMPK		208	r=0.374	<0.001*	r=0.033	0.640	r=-0.155	0.026*	-	-

[#], Kruskal-Wallis H test; [&], Mann-Whitney U test; [%], Pearson Correlation test; *, Statistically significant

Supplementary Table 2

Supplementary Table 2. Associations between pSkp2 expression with important clinic pathologic variables and pAkt, p27, and pAMPK expression.

Table 3 Univariate survival analysis

Parameters	Category	DSS			MeFS		
		No. of Case	No. of Event	p-value	No. of Case	No. of Event	p-value
Primary tumor (T)	T1	90	6	0.0942	90	10	<0.0001*
	T2	96	11		96	35	
	T3-T4	22	2		22	16	
Nodal status (N)	N0	122	7	0.0019*	122	19	<0.0001*
	N1-N2	86	12		86	42	
Stage	Stage I	69	3	<0.0001*	69	5	<0.0001*
	Stage II	115	10		115	39	
	Stage III	24	6		24	17	
pSkp LI	Low (<median)	104	3	0.0007*	104	18	0.0001*
	High (>=median)	104	16		104	43	
pAkt LI	Low (<median)	104	5	0.0109*	104	18	0.0001*
	High (>=median)	104	14		104	43	
p27 LI	High (>=median)	104	13	0.1222	104	22	0.0352*
	Low (<median)	104	6		104	39	
pAMPK LI	Low (<median)	104	5	0.0265*	104	25	0.1041
	High (>=median)	104	14		104	36	

DSS, disease-specific survival; MeFS, metastasis-free survival; *, Statistically significant

Supplementary Table 3

Supplementary Table 3. Univariate survival analysis.
